# Short birth interval and its predictors among reproductive age women in high fertility countries in sub-Saharan Africa: a multilevel analysis of recent Demographic and Health Surveys

**DOI:** 10.1186/s12884-023-05403-0

**Published:** 2023-01-30

**Authors:** Tadele Biresaw Belachew, Desale Bihonegn Asmamaw, Wubshet Debebe Negash

**Affiliations:** 1 Department of Health Systems and Policy, Institute of Public Health, College of Medicine and Health Sciences, University of Gondar, P.O.Box: 196, Gondar, Ethiopia; 2 Department of Reproductive Health, Institute of Public Health, College of Medicine and Health Sciences, University of Gondar, Gondar, Ethiopia

**Keywords:** Short birth interval, Child bearing women, Multilevel, Predictors, Sub-Saharan Africa

## Abstract

**Background:**

In developing countries, short birth interval is one of the major public health issues. It is one of the leading cause’s adverse birth outcomes in the worldwide. Despite the fact that ending maternal and perinatal morbidity and mortality is one of the Sustainable Development Goals (SDG), the burden of the problem continues to be a huge concern in developing countries, including high fertility countries. Thus, this study aimed to determine the short birth interval and its predictors in ten high fertile sub-Saharan African countries.

**Methods:**

Data for this study was obtained from the most recent Demographic and Health Surveys (DHS). A total of weighted sample of 303,979 women of childbearing age group (15– 49) who had at least two alive consecutive children was included. A multilevel mixed-effect binary logistic regression model was fitted to identify the associated factors of short birth interval. As a final step, the Adjusted Odds Ratio (AOR) was used with a confidence interval of 95% in determining statistical significance.

**Results:**

Overall prevalence of short birth interval in high fertile sub Saharan Africa was 58.74% (52.32%, 65.17%).The factors significantly associated with the short birth interval were women's educational status; primary education (AOR = 0.88; 95% CI: 0.86,0 .91), secondary and higher (AOR = 0.10; 95% CI: 0.09, 0.11), working (AOR = 0.91; 95% CI: 0.88, 0 .93), classified as rich wealth index level (AOR = 0.90; 95% CI: 0.88, 0.93),having six and above ideal number of children (AOR = 2.25; 95% CI: 2.22, 2.30), preferred waiting time two years and above to give birth (AOR = 0.83; 95% CI: 0.76, 0.89), contraceptive non users (AOR = 3.01; 95% CI: 2.93, 3.07), community level education (AOR = 1.97; 95% CI: 1.54, 2.08), rural residency (AOR = 2.17; 95% CI: 2.13, 2.22), and country Chad (AOR = 1.37; 95% CI: 1.22, 1.54).

**Conclusion:**

The prevalence of short birth interval in the top ten high fertile sub Saharan African countries is still optimally high. Therefore, the government of each country should work on the access to family planning and education in rural parts of the countries.

## Background

A short birth interval (SBI) is defined as a period of fewer than 24 months between birth and the next pregnancy, or a period of less than 33 months between births [1].In the World Health Organization (WHO), the inter-birth interval is the interval of time between two consecutive live births [2]. According to WHO reports, the interval between birth and conception should be at least 24 months in two consecutive births [3].

Using Demographic and Health Surveys (DHS) data, a comparison study of 77 countries found that having a birth interval of three or more years reduces infant mortality, maternal mortality, and young child mortality [4, 5]. It is estimated that more than 200 million women living in developing nations wish to limit or space their pregnancies, but are unable to access modern family planning methods [3, 6–9]. Dimorphic health survey studies have shown that SBI are very common in the region (Rwanda: 20%, Uganda: 25%, and Cameroon: 21%) [10].

Worldwide, birth intervals less than 18 months have been linked to higher neonatal, infant, and under-five mortality rates [5, 6, 11–13]. Similarly, women in developing regions usually have shorter spacing between births than their actual preference [14]. According to data from 52 developing countries, two-thirds of live births occurred within 30 months of the previous birth [15].

Global studies have identified several factors associated with SBI. Maternal age, maternal education level, husband education level, death of the index child, parental preference for a specific sex, no contraception use, ideal number of children, socio-cultural factors, religion, short nursing duration (less than 24 months), and poverty levels are just a few of them [15–22] and ANC follow up [23].

The 2030 Sustainable Development Goals (SDGs) aim to ensure healthy lives and promote wellbeing for all people by combining multi-system solutions at global, regional, and national levels [24]. One of these goals is to lower the neonatal mortality rate to less than 12 per 1,000 live births [25, 26] which is at a stable stage in developing countries.

Despite the implementation of many global and national initiatives and treatments aimed at reducing the burden of children under the age of five, newborn and neonatal death rates continue to rise [27, 28], SBI remains one of the leading causes of child mortality [5, 22] in developing countries [27, 28].

However, specific African countries have studied the predictors of SBI among reproductive-age women, including Ethiopia [28–30] Uganda [31] and Uganda and Zimbabwe [21]. There have been no studies that have combined specific high fertility countries in SSA to understand why they have such high SBI. In addition, these previous studies failed to consider community-level factors and their interaction with individual-level factors. Multilevel approaches will provide an understanding of factors affecting SBI at both the individual and community levels.

Policymakers, program planners, and other stakeholders will benefit from the findings of this study, which will help them develop and implement effective measures to avoid SBIs in Sub-Saharan African countries. As a result, the goal of this study was to look at both individual and community-level predictors of SBI among women in Sub-Saharan Africa's top ten high-fertility countries.

## Methods

### Study settings and data source

The study was a cross-sectional assessment of data from recent Demographic and Health Surveys (DHSs) conducted between January 2010 and December 2018 of ten countries in SSA. As a result of high fertility rates in some countries, the interval between births can be short, causing poor fetal and maternal health outcomes [32–34]. So, our study examined the time between the deliveries of one child to the delivery of the next child in top ten high fertility sub Saharan African countries (Niger, Democratic Republic Congo, Mali, Chad, Angola, Burundi, Nigeria, Gambia, and Burkina Faso were included in this study). These countries were selected because they are the top ten countries with high fertility rates in SSA with fertility rates above 5.0, a higher value than the rate of 4.44 in SSA and 2.47 worldwide [35]. One country (Somalia) with no DHS data was excluded from the analysis. The data for these countries were obtained from the official database of the DHS program, www.measuredhs.com after authorization was allowed via online request by explaining the purpose of our study. We used the woman record (BR file) data set and extracted the dependent and independent variables. The DHS is a nationally representative household survey that uses face-to-face interviews on a wide range of population, health, nutrition tracking, and effect assessment measures. A two-stage stratified sampling procedure was used to identify study participants. In the first step, enumeration areas (EAs) were chosen at random, while households were chosen in the second stage [36]. The current study included individual-level data for 303,979 married women who had at least two live births during the five years preceding years. Women who had never married were not included in the study (Table 1).

**Table 1:** Description of Surveys and sample size characteristics in high fertility countries in SSA (*n* = 303,979)

Countries	Survey year	Weighted sample(n)	Weighted percentage (%)
Angola	2015–16	4991	1.64
Burkina Faso	2010	39,053	12.85
Burundi	2016–17	23,563	7.75
Chad	2014–15	44,311	14.58
DR Congo	2013–14	28,559	9.40
Gambia	2013	19,209	6.32
Mali	2018	25,279	8.32
Nigeria	2012	84,226	27.71
Niger	2012	34,790	11.44

### Study variables

#### Dependent variable

In this study, the outcome variable was a SBI, which was dichotomized into "yes = 1" and "no = 0". A SBI is defined as an interval of less than 33 months between two successive live births. A preceding birth interval greater than 33 months was defined as a non-SBI, in accordance with WHO recommendations [37]. The birth interval was calculated by subtracting the birth date of the first child from the date of the second child [38].

#### Independent variables

All of the independent variables were chosen after a thorough examination of the literature [28, 31, 39–41] and individual-level factors and community-level variables were used to categorize the independent variables. Individual-level variables were age at first marriage, educational status of respondents (no formal education, primary education, secondary and above), occupation (not working, working), wealth status, media exposure, ideal number of children (less than 6 years, 6 years and above), husband education (no formal education, primary education, secondary and above), and husband occupation (not working, working), preferred waiting time to birth (less than 2 years, 2 years and above), and contraceptive use (yes, no). Of the community level variables, residence (rural, urban) were directly accessed from DHS data sets. However, community level poverty (low, high) and community level education (uneducated, educated) were constructed by aggregating individual-level characteristics at the cluster level [42–44]. They were classified as high or low based on the distribution of proportion values generated for each community after using the histogram to check the distribution. Because the aggregate variable was not normally distributed, the median value was chosen as a classification cut-off point.

#### Wealth status

The variable wealth index was re-categorized as “Poor”, “Middle”, and “Rich” categories by merging poorest with poorer and richest with richer [42, 45, 46].

#### Media exposure

Media exposure was calculated by aggregating TV watching, radio listening, and reading newspapers and woman who has exposure to either of the media sources was categorized as having media exposure and the rest considered as having no media exposure [30].

#### Preferred waiting time to birth

Categorized as those who wish to wait 2 years and above and those who wish to wait less than 2 years before another pregnancy [28].

#### Ideal number of children

Categorized into those who need six or more children and those who need fewer than six [28].

### Data analysis

For data analysis Stata version 16 software was used. Throughout the analyses, sampling weight was used to adjust for the unequal probability of sample selection and the differences in response rates. Before data analysis, the data were weighted to ensure that the DHS sample was representative and to provide reliable estimates and standard errors.

Due to the hierarchical nature of the DHS data (i.e., mothers are nested inside clusters), a multivariable multilevel logistic regression analysis was used to estimate the effects of each SBI predictor.

The equation used for fitting the multilevel logistic regression model was as follows:

$$\mathrm{Log}\;\left[\mathrm{\pi}{ij}\;/\;\left(1-\mathrm{\pi}{ij}\right)\right]\;=\;\mathrm\beta0+\;\mathrm\beta1\mathrm{xij}+\mathrm\beta2\mathrm{xij}\;\dots+\;\mathrm \mu0\mathrm j\;+\;\mathrm e0\mathrm{ij}$$

Where, πiϳ: the probability of short birth interval, 1- πiϳ: the probability of no short birth interval, β1xiϳ: individual and community level variables for the i^th^ individual in group j, respectively. The ß’s are fixed coefficients indicating a unit increase in X can cause a ß unit increase in probability short birth interval. While the ß_0_ is intercept that is the effect on short birth interval when the effect of all explanatory variables are absent. The u0j shows the random effect (effect of the community on the women’s short birth interval) for the j^th^ community [47, 48].

Four models were fitted in this study. Model 0 (Empty model) was used to assess random variability in the intercept and determine the intra-class correlation coefficient (ICC) and Proportion Change in Variance (PCV). Model I assessed the effects of individual-level predictors. Model II explored the effects of community-level predictors, while Model III (Full model) investigated the effects of both individual and community-level features at the same time. Model III was the best-fitted model since it had the lowest deviance. Variables having a p-value less than 0.2 in bivariable analysis were used for multivariable analysis [49–51]. Finally, in the multivariable analysis, adjusted odds ratios with 95% confidence intervals and a p-value of less than 0.05 were used to identify factors of SBI.

## Results

### Individual level factors

Out of the total respondents, 202,348 (66.57%) women were not attended formal education, of the study participants 67,652 (23.77%) were not working, and 176,471 (58.25%) of the respondents had media exposure towards birth spacing. Among the participants, 92,383 (58.33%) had preferred two and above years waiting time to birth. With regard to their economic status, 134,243 (44.16%) women were from households under the poor wealth quintiles and 105,246 (34.62%) were from the rich wealth quintiles (Table 2).

**Table 2:** Individual characteristics of respondents in high fertile countries in sub-Saharan Africa (*n* = 303,979)

Variables	Categories	Frequency	Percentage (%)
Age at birth of index child	< 18	175,305	57.67
	19–24	109,943	36.17
	≥ 25	18,732	6.16
Educational status of respondents	No formal education	202,348	66.57
	Primary education	82,226	27.05
	Secondary and above	19,407	6.38
Husband education	No formal education	180,514	59.43
	Primary education	78,558	25.86
	Secondary and higher	44,651	14.70
Occupation of respondents	Not working	67,652	23.77
	Working	216,949	76.23
Husband occupation	Not working	14,159	4.67
	Working	289,148	95.33
Wealth index	Poor	134,243	44.16
	Middle	64,491	21.22
	Rich	105,246	34.62
Media exposure	No	126,508	41.75
	Yes	176,471	58.25
Ideal number of children	Less than 6	80,531	26.49
	6 and above	223,448	73.51
Preferred waiting time to birth	Less than 2 years	65,995	41.67
	2 and above years	92,383	58.33
Contraceptive use	Yes	49,717	16.36
	No	254,263	83.64

### Community level factors

Of the study participants 226,296 (74.44%) were resides in rural area. Of the respondents 155,006 (50.99%) women were from communities with low proportion of poverty level. Among respondents 202,347 (66.57%) were under low proportion of community education level (Table 3).

**Table 3:** Community level characteristics of respondents in high fertile countries in sub-Saharan Africa (*n* = 303,979)

Variables	Categories	Frequency	Percentage (%)
Residence	Urban	77,684	25.56
	Rural	226,296	74.44
Community-level poverty	Low	155,006	50.99
	High	148,962	49.01
Community education	Uneducated	202,347	66.57
	Educated	101,633	33.43
Country	Angola	4991	1.64
	Burkina Faso	39,053	12.85
	Burundi	23,563	7.75
	Chad	44,311	14.58
	DR Congo	28,559	9.40
	Gambia	19,209	6.32
	Mali	25,279	8.32
	Nigeria	84,226	27.71
	Niger	34,790	11.44

### Prevalence of short birth interval in top ten high fertile sub-Saharan African countries

**Fig. 1:**
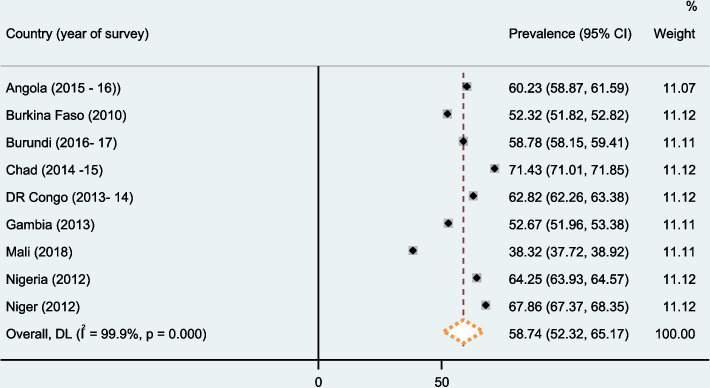
Forest plot of overall prevalence of short birth interval in top ten high fertile Sub-Saharan countries. Source: Authors’ computations

Overall, the prevalence of SBI in top ten high fertile sub Saharan Africa countries was 58.74% (52.32%, 65.17%). The prevalence of SBI was ranged from 38.32% in Mali to 71.43% in Chad (Fig. 1).

### Predictors of short birth interval

In table four, the study showed that women with Secondary and higher educational status were 0.9 times less likely to have SBI than those who had no formal education (AOR = 0.10; 95% CI: 0.09, 0.11) and those women with primary education level were 0.12 times less likely to have SBI compared to those with no formal education (AOR = 0.88; 95% CI: 0.86,0 0.91). Women who had employed were 0.09 times less likely to have SBI compared to those unemployed (AOR = 0.91; 95% CI: 0.88, 0 0.93). The odds of SBI in the rich wealth index level were 0.1 times less likely to be happened compared to those women in the poor level (AOR = 0.90; 95% CI: 0.88, 0.93). The likelihood of women having SBI was high among women who had six and above (AOR = 2.25; 95% CI: 2.22, 2.30) ideal number of children in comparison to their counterparts. Women with two years and above preferred waiting time to birth were 0. 17 lower chance of having SBI (AOR = 0.83; 95% CI: 0.76, 0.89) against those who wanted a wait time of less than two years**.** Women who did not use contraception were also three times more likely to have a SBI (AOR = 3.01; 95% CI: 2.93, 3.07) compared to those had used contraceptives.

With regard to the community level factors, women classified as low Community level education were more likely to had risk of SBI (AOR = 1.97; 95% CI: 1.54, 2.08) than high Community level education. Women in rural areas had a 2.17 times higher chance of having a SBI than those in urban areas. (AOR = 2.17; 95% CI: 2.13, 2.22). Furthermore, women in Chad were 1.37 times more likely (AOR = 1.37; 95% CI: 1.22, 1.54) to have higher probability of SBI compared to women in the Angola (Table 4).

**Table 4:** multivariable analyses for factors affecting short birth interval **(***n* = 303,979)

**Variables**	Short birth interval		Model 0	Model 1 AOR (95% CI)	Model 2 AOR (95%CI)	Model 3 AOR (95%CI)
	No	yes				
**Individual level Characteristics**						
Educational status of the respondents						
No formal education	72,570	129,776		1		1
Primary education	32,450	49,728		0.94 (0.92, 0.97)*		0.88(0.86,0 .91)*
Secondary and higher	8395	11,012		0.96 (0.91, 1.01)		0.10 (0.09,0.11)*
Occupation of respondents						
Not working	23,173	44,479		1		1
Working	84,058	132,891		0.83 (0.81, 0.85)*		0.91 (0.88, 0.93)*
Wealth index						
Poor	47,572	86,672		1		1
Middle	23,707	40,784		0.98 (0.95, 1.01)		0.96 (0.93, 0.99)
Rich	42,184	63,061		0.90 (0.88, 0.93)*		0.90 (0.88, 0.93)*
Media exposure						
No	43,482	83,025		1		1
Yes	69,683	106,788		0.85 (0.83, 0.87)*		1.01 (0.98, 1.04)
Ideal number of children						
Less than 6	35,011	45,520		1		1
6 and above	78,452	144,997		1.41 (1.37, 1.45)*		2.25 (2.22, 2.30)*
Preferred waiting time to birth						
Less than 2 years	24,378	41,617		1		1
2 and above years	34,099	58,284		1.02 (0.99, 1.05)		0.83 (0.76, 0.89)*
Contraceptive use						
Yes	20,244	29,473		1		1
No	93,219	161,044		1.01 (0.98, 1.05)		3.01 (2.93, 3.07)*
Community level variables						
Community level education						
Educated	72,570	129,776			1	1
uneducated	40,892	60,741			1.13 (1.08, 1.17)*	1.97 (1.54, 2.08)*
Community level Poverty						
Low	59,861	95,145			1	1
High	53,597	95,365			1.06 (1.02, 1.10)*	1.00 (0.96, 1.04)
Residency						
Urban	32,603	45,080			1	1
Rural	80,860	145,436			1.26 (1.24, 1.29)*	2.17 (2.13, 2.22)*
Country						
Angola	1985	3006			1	1
Burkina Faso	18,620	20,433			0.66 (0.62, 0.71)*	0.59 (0.53, 1.01)
Burundi	9712	13,851			0.87 (0.81, 0.92)*	0.93 (0.83, 1.05)
Chad	12,660	31,651			1.57 (1.47, 1.67)*	1.37 (1.22, 1.54)*
DR Congo	10,619	17,940			1.08 (1.01, 1.15)*	1.08 (0.96, 1.21)
Gambia	9092	10,117			0.79 (0.74, 0.85)*	0.70 (0.63, 1 .12)
Mali	9482	15,797			1.05 (0.98, 1.12)	0.91 (0.81, 1.03)
Nigeria	30,110	54,114			1.15 (1.08, 1.22)*	1.10 (0.98, 1.23)
Niger	11,182	23,607			1.30 (1.22, 1.39)*	1.09 (0.97, 1.22)
Random effect results			Model 0	Model 1	Model 2	Model 3
Variance (%)			61.35	47.29	17.34	11.32
ICC (%)			14.76	14.01	11.28	9.50
MOR			20.25	17.78	10.76	8.70
PCV			Ref	22.9	71.7	81.5
Deviance(-2LLR)			159,672	143,450	134,576	113,268

Null model: adjusted for individual-level characteristics

Model 2: Adjusted for community-level characteristics

Model 3: adjusted for both individual and community level characteristics

*AOR* Adjusted Odds Ratio, *COR* Crude Odds Ratio

^*^ Statistically significant at *p*-value < 0.05

## Discussion

In this study, we assessed the prevalence and predictors of SBI in ten high fertility Sub-Saharan African countries using the DHS dataset from 2010 to 2018. This study revealed that residence site, women’s educational status, women’s occupation, wealth index, contraceptive use, ideal number of children; preferred waiting time, community level poverty, and country were the independent predictors of SBIs.

This study revealed the prevalence of SBI was 58.74% (95% CI: 52.32%, 65.17%).This finding is consistent with studies conducted in Uganda (52.4%) [31], Ethiopia [9, 52], Kassala eastern Sudan (60.6%) [53]. However, the finding is higher than studies conducted in Ethiopia [12, 18, 19, 28, 39, 54], Ghana (49.7%) [55], Tanzania (48.8%) [6], Iran (28.8%) [56] and rural Bangladesh (24.6%) [1]. This could be explained by the prior studies' small sample sizes and social and cultural differences between the current study and previous studies. In this study, women with primary and secondary education were 12% and 90% less likely to have SBI than those without formal education. This discovery is in line with the findings of Tanzanian [6], Democratic Republic of Congo [10], Ethiopia [16, 22, 52], and Kassala, Eastern Sudan [53]. The reason might be if the women's education level increases, they will also be more knowledgeable and aware of the effects of short childbirth intervals on maternal and child health [57]. As a result, women with a primary, secondary, or higher education are less likely to have SBI than women with no education.

Women with occupation were 9 percent less likely to have SBI than not with occupation women. This might be the likelihood of SBI among women who are employed may be explained by the relatively busy lifestyle and awareness of contraceptives utilization, which may help women to reduce their childbearing period [16, 29].

Women from the rich households were less likely to experience SBI compared with women from the poor household. This study is in line with previous research [22, 29]. This could be because women with more resources have easier access to health information and education, resulting in longer birth intervals.

The study found that women with a preferred waiting time of two years or more had a 17% lower chance of having SBI than women with a preferred waiting time of less than two years. This might be due to the fact that women who preferred to have space to give birth will use contraceptive methods [58].

Moreover, women who have a desire to have six or more children had 2.25 times greater risk of SBI compared to those with a desire of fewer than six children. In the developing countries of Sub-Saharan Africa, parents and their community members have a desire for more children because of socio-cultural and religious interests. This result is also supported by research undertaken in Ethiopia [16, 39] and Sudan [53]. Furthermore, in the developing world, livestock is the sole source of livelihood, where more children are considered beneficial to have more keepers for their cattle. This perception of the developing community towards more children has contributed to the SBI in these regions of sub Saharan Africa [59].

In addition Contraceptive use has been linked to closed birth intervals. Women who had no utilized contraceptive had 3 times greater risk of SBI compared to those had used. This study also supported with several studies conducted in Ethiopia [18, 39, 54] and Nigeria [60]. This may be because contraceptive use delays a birth interval and limits fertility as it affects the reproductive process [61]. Women who do not use contraceptives may also miss the opportunity to contact a healthcare provider, causing them to be less aware of the outcome of SBI.

Women living in rural areas have a 2.17 times higher risk of SBI than those living in urban areas. Similar findings were found in Ethiopia [9] and Democratic Republic of Congo [10]. The reason may be that women living in rural areas are socio-economically disadvantaged [1] and inaccessible to modern contraceptive methods.

According to this study, women who live in communities with a low proportion of educational level were more likely to have risk of SBI compared to women who lived in a community of high educational level. The reason might be if education level increases, they will also be more knowledgeable and aware of the effects of short childbirth intervals on maternal and child health [62].

Moreover, women living in Chad were 1.37 times more likely to have risk of SBI compared to women in the Angola. This could be due to the difference in wealth status of the women for under low income countries of Chad that leads inaccessibility of maternal health services such as family planning services and access to health information [63].

The study's strength was its use of national survey data sets from many countries. Due to the cross-sectional nature of the data, this study may not demonstrate cause and effect relationship. Variation of time of data sets that the latest data sets can have overestimated the prevalence of SBI in this study. In addition, this study did not include variables such as ANC visits, types of marriage, which may have a significant impact on birth spacing.

## Conclusion

The prevalence of SBI in the top ten high fertile sub Saharan African countries is still optimally high. In the multilevel multivariable logistic regression model; residence, women’s education, occupation, wealth index, contraceptive use, ideal number of children, preferred waiting time, community level poverty, and country were the independent predictors of SBI in the top ten high fertile sub Saharan African countries. As a result, each country's government should endeavor to improve access to family planning and education in rural areas.

## Data Availability

Data for this study were sourced from Demographic and Health surveys (DHS), which is freely available online at (https://dhsprogram.com).

## References

[1] De Jonge HC, Azad K, Seward N, Kuddus A, Shaha S, Beard J (2014). Determinants and consequences of short birth interval in rural Bangladesh: a cross-sectional study. BMC Pregnancy Childbirth.

[2] Conde-Agudelo A, Rosas-Bermudez A, Castaño F, Norton MH (2012). Effects of birth spacing on maternal, perinatal, infant, and child health: a systematic review of causal mechanisms. Stud Fam Plann.

[3] World Health Organization. Report of a WHO technical consultation on birth spacing: Geneva, Switzerland 13-15 June 2005. World Health Organization; 2007.

[4] Rutstein S. Effect of birth intervals on mortality and health: multivariate cross country analyses. In: Conference on Optimal Birth Spacing for Central America, Antigua, Guatemala 2003 Jun, Vol. 2003.

[5] Molitoris J, Barclay K, Kolk M (2019). When and where birth spacing matters for child survival: an international comparison using the DHS. Demography.

[6] Exavery A, Mrema S, Shamte A, Bietsch K, Mosha D, Mbaruku G (2012). Levels and correlates of non-adherence to WHO recommended inter-birth intervals in Rufiji. Tanzania BMC pregnancy and childbirth.

[7] Memirie ST, Dagnaw WW, Habtemariam MK, Bekele A, Yadeta D, Bekele A, et al. Addressing the impact of noncommunicable diseases and injuries (NCDIs) in Ethiopia: findings and recommendations from the Ethiopia NCDI Commission. Ethiop J Health Sci. 2022;32(1). 10.4314/ejhs.v32i1.18 PMC8864405 35250228

[8] Abdel-Fattah M, Hifnawy T, El Said TI, Moharam MM, Mahmoud MA (2007). Determinants of birth spacing among Saudi women. J Fam Community Med.

[9] Yohannes S, Wondafrash M, Abera M, Girma E (2011). Duration and determinants of birth interval among women of child bearing age in Southern Ethiopia. BMC Pregnancy Childbirth.

[10] Chirwa TF, Mantempa JN, Kinziunga FL, Kandala JD, Kandala N-B (2014). An exploratory spatial analysis of geographical inequalities of birth intervals among young women in the Democratic Republic of Congo (DRC): a cross-sectional study. BMC Pregnancy Childbirth.

[11] Tigabu S, Demelew T, Seid A, Sime B, Manyazewal T (2018). Socioeconomic and religious differentials in contraceptive uptake in western Ethiopia: a mixed-methods phenomenological study. BMC Womens Health.

[12] Dibaba Y. Child spacing and fertility planning behavior among women in mana district, Jimma Zone, South West Ethiopia. Ethiop J Health Sci. 2010;20(2). 10.4314/ejhs.v20i2.69433 PMC3275838 22434965

[13] Brhane M, Hagos B, Abrha MW, Weldearegay HG (2019). Does short inter-pregnancy interval predicts the risk of preterm birth in Northern Ethiopia?. BMC Res Notes.

[14] Abdel-Tawab NG, Loza S, Zaki A (2008). Helping Egyptian women achieve optimal birth spacing intervals through fostering linkages between family planning and maternal/child health services.

[15] Rutstein SO (2005). Effects of preceding birth intervals on neonatal, infant and under-five years mortality and nutritional status in developing countries: evidence from the demographic and health surveys. Int J Gynecol Obstet.

[16] Begna Z, Assegid S, Kassahun W, Gerbaba M (2013). Determinants of inter birth interval among married women living in rural pastoral communities of southern Ethiopia: a case control study. BMC Pregnancy Childbirth.

[17] Gemmill A, Lindberg LD (2013). Short interpregnancy intervals in the United States. Obstet Gynecol.

[18] Tessema GA, Zeleke BM, Ayele TA (2013). Birth interval and its predictors among married women in Dabat District, Northwest Ethiopia: a retrospective follow up study. Afr J Reprod Health.

[19] Shallo S, Gobena T. Duration of birth interval and associated factors among married women in Dodota Woreda, Arsi Zone, Ethiopia. J Health Educ Res Dev. 2019;7(1).

[20] Nega W, Woncheko E. The determinants of birth interval in rural Ethiopia. 2016.

[21] McGuire C, Stephenson R (2015). Community factors influencing birth spacing among married women in Uganda and Zimbabwe. Afr J Reprod Health.

[22] Hailu D, Gulte T. Determinants of short Interbirth interval among reproductive age mothers in Arba Minch District Ethiopia. Int J Reprod Med. 2016;2016. 10.1155/2016/6072437 PMC4863097 27239553

[23] Mihretie GN, Yenealem Beyene F, Getnet Kassa B, Degu Ayele A, Muche Liyeh T, Minuye BB (2021). Determinants of short birth interval among women in South Gondar, Ethiopia: community-based unmatched case-control study. Archives of Public Health.

[24] Organization WH (2019). Snakebite envenoming: a strategy for prevention and control.

[25] Lee BX, Kjaerulf F, Turner S, Cohen L, Donnelly PD, Muggah R (2016). Transforming our world: implementing the 2030 agenda through sustainable development goal indicators. J Public Health Policy.

[26] Manyazewal T (2017). Using the World Health Organization health system building blocks through survey of healthcare professionals to determine the performance of public healthcare facilities. Arch Public Health.

[27] Organization WH (2015). WHO recommendations on interventions to improve preterm birth outcomes.

[28] Aychiluhm SB, Tadesse AW, Mare KU, Abdu M, Ketema A (2020). A multilevel analysis of short birth interval and its determinants among reproductive age women in developing regions of Ethiopia. PLoS ONE.

[29] Shifti DM, Chojenta C, G Holliday E, Loxton D (2020). Individual and community level determinants of short birth interval in Ethiopia: a multilevel analysis. PloS one..

[30] Tesema GA, Worku MG, Teshale AB (2021). Duration of birth interval and its predictors among reproductive-age women in Ethiopia: Gompertz gamma shared frailty modeling. PLoS ONE.

[31] Aleni M, Mbalinda SN, Muhindo R. Birth intervals and associated factors among women attending young child clinic in Yumbe Hospital, Uganda. Int J Reprod Med. 2020;2020. 10.1155/2020/1326596 PMC6964709 31984212

[32] Bauserman M, Nowak K, Nolen TL, Patterson J, Lokangaka A, Tshefu A (2020). The relationship between birth intervals and adverse maternal and neonatal outcomes in six low and lower-middle income countries. Reprod Health.

[33] Zimicki S. The relationship between fertility and maternal mortality. Contraceptive use and controlled fertility, health issues for women and children. 1989.

[34] Winikoff B. The effects of birth spacing on child and maternal health. Studies Fam Plann. 1983:231–45. 6648993

[35] African countries with the highest fertility rate | Statista https://worldpopulationreview.com/countries/total-fertility-rate. Cited on 8 Dec 2021.

[36] Corsi DJ, Neuman M, Finlay JE, Subramanian S (2012). Demographic and health surveys: a profile. Int J Epidemiol.

[37] WHO Expert Committee on Biological Standardization. Meeting, World Health Organization. WHO expert committee on biological standardization: fifty-sixth report. World Health Organization; 2007.

[38] Rutstein SO. Trends in birth spacing. DHS comparative reports no 28. Calverton: ICF Macro; 2011.

[39] Gebrehiwot SW, Abera G, Tesfay K, Tilahun W (2019). Short birth interval and associated factors among women of child bearing age in northern Ethiopia, 2016. BMC Womens Health.

[40] Roble AK, Osman MO, Ibrahim AM, Wedajo GT, Abdi US (2021). Determinants of short birth interval among ever married reproductive age women living in Jigjiga, Eastern Ethiopia 2020 (unmatched case–control study). SAGE Open Med.

[41] Nausheen S, Bhura M, Hackett K, Hussain I, Shaikh Z, Rizvi A (2021). Determinants of short birth intervals among married women: a cross-sectional study in Karachi, Pakistan. BMJ Open.

[42] Kefale B, Yalew M, Damtie Y, Adane B (2020). A multilevel analysis of factors associated with teenage pregnancy in Ethiopia. Int J Women's Health.

[43] Ahinkorah BO (2020). Predictors of unmet need for contraception among adolescent girls and young women in selected high fertility countries in sub-Saharan Africa: a multilevel mixed effects analysis. PLoS ONE.

[44] Belay DG, Aragaw FM, Teklu RE, Fetene SM, Negash WD, Asmamaw DB, et al. Determinants of inadequate minimum dietary diversity intake among children aged 6–23 months in sub-Saharan Africa: pooled prevalence and multilevel analysis of demographic and health survey in 33 sub-Saharan African Countries. Front Nutr. 2022;9. 10.3389/fnut.2022.894552 PMC9284213 35845763

[45] Shagaro SS, Gebabo TF, Mulugeta BT (2022). Four out of ten married women utilized modern contraceptive method in Ethiopia: a multilevel analysis of the 2019 Ethiopia mini demographic and health survey. Plos one.

[46] Birhanu BE, Kebede DL, Kahsay AB, Belachew AB (2019). Predictors of teenage pregnancy in Ethiopia: a multilevel analysis. BMC Public Health.

[47] Merlo J, Chaix B, Yang M, Lynch J, Råstam L (2005). A brief conceptual tutorial on multilevel analysis in social epidemiology: interpreting neighbourhood differences and the effect of neighbourhood characteristics on individual health. J Epidemiol Community Health.

[48] Tessema ZT, Teshale AB, Tesema GA, Yeshaw Y, Worku MG (2021). Pooled prevalence and determinants of modern contraceptive utilization in East Africa: a multi-country analysis of recent demographic and health surveys. PLoS ONE.

[49] Negash WD, Fetene SM, Shewarega ES, Fentie EA, Asmamaw DB, Teklu RE (2022). Multilevel analysis of quality of antenatal care and associated factors among pregnant women in Ethiopia: a community based cross-sectional study. BMJ Open.

[50] Worku MG, Tessema ZT, Teshale AB, Tesema GA, Yeshaw Y (2021). Prevalence and associated factors of adolescent pregnancy (15–19 years) in East Africa: a multilevel analysis. BMC Pregnancy Childbirth.

[51] Aalmneh TS, Alem AZ, Tarekegn GE, Kassew T, Liyew B, Terefe B (2022). Individual and community-level factors of abortion in East Africa: a multilevel analysis. Archives of Public Health.

[52] Ayane GB, Desta KW, Demissie BW, Assefa NA, Woldemariam EB (2019). Suboptimal child spacing practice and its associated factors among women of child bearing age in Serbo town, JIMMA zone Southwest Ethiopia. Contracept Reprod Med.

[53] Ali AA, Yassin K, Ramadan N (2014). Determinant of inter-pregnancy birth interval in Kassala Eastern Sudan. Curr Women's Health Rev.

[54] Ejigu AG, Yismaw AE, Limenih MA (2019). The effect of sex of last child on short birth interval practice: the case of northern Ethiopian pregnant women. BMC Res Notes.

[55] Alhassan AR, Anyinzaam-Adolipore JN, Abdulai K (2022). Short birth interval in Ghana: Maternal socioeconomic predictors and child survival. Popul Med.

[56] Fallahzadeh H, Farajpour Z, Emam Z (2013). Duration and determinants of birth interval in Yazd, Iran: a population study. Iran J Reprod Med.

[57] Basu AM, Stephenson R (2005). Low levels of maternal education and the proximate determinants of childhood mortality: a little learning is not a dangerous thing. Soc Sci Med.

[58] Bawah AA, Akweongo P, Simmons R, Phillips JF. Women's fears and men's anxieties: the impact of family planning on gender relations in northern Ghana. Wiley Online Library; 1999. 10.1111/j.1728-4465.1999.00054.x 10216896

[59] De Haan LJ (2000). Globalization, localization and sustainable livelihood. Sociol Rural.

[60] Dim C, Ugwu E, Iloghalu E (2013). Duration and determinants of inter-birth interval among women in Enugu, south-eastern Nigeria. J Obstet Gynaecol.

[61] Rahman M, DaVanzo J (1993). Gender preference and birth spacing in Matlab Bangladesh. Demography.

[62] Frost MB, Forste R, Haas DW (2005). Maternal education and child nutritional status in Bolivia: finding the links. Soc Sci Med.

[63] Ewerling F, Victora CG, Raj A, Coll CV, Hellwig F, Barros AJ (2018). Demand for family planning satisfied with modern methods among sexually active women in low-and middle-income countries: who is lagging behind?. Reprod Health.

